# A Single Oral Vitamin D_3_ Bolus Reduces Inflammatory Markers in Healthy Saudi Males

**DOI:** 10.3390/ijms231911992

**Published:** 2022-10-09

**Authors:** Shareefa A. AlGhamdi, Nusaibah N. Enaibsi, Hadeil M. Alsufiani, Huda F. Alshaibi, Sawsan O. Khoja, Carsten Carlberg

**Affiliations:** 1 Department of Biochemistry, Faculty of Sciences, King Abdulaziz University, Jeddah 21589, Saudi Arabia; 2 Vitamin D Pharmacogenomics Research Group, King Abdulaziz University, Jeddah 21589, Saudi Arabia; 3 Experimental Biochemistry Unit, King Fahd Medical Research Center, King Abdulaziz University, Jeddah 21589, Saudi Arabia; 4 Institute of Animal Reproduction and Food Research, Polish Academy of Sciences, 10-748 Olsztyn, Poland; 5 Institute of Biomedicine, School of Medicine, University of Eastern Finland, 70211 Kuopio, Finland

**Keywords:** vitamin D_3_ supplementation, vitamin D bolus, inflammatory cytokines

## Abstract

Vitamin D deficiency has increased in the general population and is a public health issue. Vitamin D plays an important role in regulating the immune system, e.g., by modulating the production of inflammatory cytokines. In most countries, the recommended maximal daily dose of vitamin D_3_ is 4000 IU (100 µg) per day. In this study, we investigated whether a single vitamin D_3_ bolus can reduce the levels of the inflammatory markers interleukin (IL) 6, IL8 and tumor necrosis factor (TNF) within one month. Fifty healthy Saudi males were recruited from the local community in Jeddah city and were orally supplemented with a single dose of 80,000 IU vitamin D_3_. Serum samples were collected at time points 0, 1 and 30 days, and serum levels of IL6, IL8 and TNF, parathyroid hormone (PTH), 25-hydroxyvitamin D_3_ (25(OH)D_3_), triglycerides, cholesterol, calcium (Ca^2+^) and phosphate (PO_4−_) were determined. On average, the vitamin D_3_ bolus resulted in a significant increase in vitamin D status as well as in a significant decrease in the levels of inflammatory cytokines even one month after supplementation without changing serum Ca^2+^, PO_4−_ or lipid levels. In conclusion, single high-dose vitamin D_3_ supplementation is safe for reducing inflammation markers and may lead to an update of current recommendations for vitamin D intake, in order to prevent critical health problems.

## 1. Introduction

Inflammation is primarily the reaction of the innate immune system to microbial and non-microbial molecules, referred to as pathogen- and damage-associated molecular patterns, such as surface molecules of bacteria or nucleic acids of viruses, noxious chemicals or physical trauma [1]. Acute inflammation is part of the normal protective response of the host and is resolved after 1–2 weeks [2]. In contrast, in the case of chronic inflammation lasting months, years or even decades, the host is unable to resolve the cause of the response, which is in most cases non-microbial molecules, e.g., deriving from a lipid overload [3]. This often results in the onset of autoinflammatory or autoimmune disorders [4]. Moreover, most chronic diseases such as type 2 diabetes, obesity, neurodegenerative diseases, cancer and arteriosclerosis are associated with chronic inflammation. Since cancer and cardiovascular diseases are the main causes of mortality in most countries of the world, chronic inflammation significantly reduces health span and needs to be avoided [5,6,7].

There are a number of different, effective anti-inflammatory medications including both steroidal and nonsteroidal anti-inflammatory drugs. These drugs have a high therapeutic efficacy toward acute inflammation; however, they are not effective in curing or treating chronic inflammatory diseases, such as inflammatory bowel disease (Crohn’s disease and ulcerative colitis), osteoarthritis, bronchiectasis and rheumatoid arthritis [7]. Due to the undesirable side effects associated with nonsteroidal anti-inflammatory drugs and steroidal medications, an increased interest has been prompted for natural compounds, including herbal remedies and dietary supplements, as these have been used for centuries to reduce inflammation and pain [8]. Thus, changes in lifestyle and diet help to minimize the effects of inflammation and its stimulators.

Vitamin D deficiency is associated with a variety of illnesses, many of which are linked to inflammation [9,10]. The multiple effects of vitamin D_3_ on the immune response suggest that the molecule might be a promising candidate for treating various immune-related disorders. Clinical studies have shown that vitamin D_3_ plays an important role in regulating the innate immune response against different pathogens [11]. In addition, the adaptive immune response in a variety of inflammatory and autoimmune diseases can be regulated by vitamin D_3_ [12]. These findings suggest the beneficial effects of vitamin D_3_ supplementation on reducing the risk and adverse consequences of inflammatory diseases. A number of studies have investigated how vitamin D_3_ reduces the risk of viral infection [13,14,15]. For example, low levels of vitamin D_3_ are associated with the release of major proinflammatory cytokines, such as IL6 [16]. The major challenges of severe forms of SARS-CoV-2 infections are cytokine storms, in which innate immunity loses control and produces excessive amounts of cytokines such as IL6 and TNF [17]. Vitamin D_3_ supplementation can inhibit the progression of COVID-19, while vitamin D deficiency promotes acute dyspnea [18]. Thus, there are many reasons to raise vitamin D status, defined by the serum 25(OH)D_3_ concentrations, of the general population.

Vitamin D_3_ supplementation is mostly performed in the form of daily tablets with doses between 400 and 4000 IU (10–100 µg), but the lower doses used are often inadequate, and adherence to daily intake is not optimal [19]. Thus, a monthly bolus may a convenient alternative to daily supplementation [20]. Nevertheless, there is a lack of systematic evaluation of high-dose vitamin D treatments. Individuals with an initial 25(OH)D_3_ serum level of less than 50 nM responded very well to a vitamin D_3_ bolus of 500,000 IU, as their vitamin D status increased to an optimal level of more than 100 nM [21,22,23]. In contrast, a monthly dose of 50,000 IU vitamin D_3_ takes about 3 months to reach satisfactory 25(OH)D_3_ levels [24]. In the vitamin D intervention studies VitDbol (https://clinicaltrials.gov/ct2/show/NCT02063334, accessed on 20 July 2022) and VitDHiD (https://clinicaltrials.gov/ct2/show/NCT03537027, accessed on 20 July 2022), a single vitamin D_3_ bolus of 80,000 IU was proven to be a safe treatment for healthy individuals in Finland [25,26,27,28]. Therefore, we chose the same protocol for investigating the effect of a single vitamin D_3_ bolus on the inflammatory markers IL6, IL8 and TNF among healthy male subjects from Saudi Arabia.

## 2. Results

### 2.1. Characteristics of the Participants

The demographic characteristics of the participants (Table 1) indicate a mean age of 30.3 years, a mean body mass index (BMI) of 27.6 kg/m^2^ and a mean WHR (waist-to-hip ratio) of 0.91. Thus, 66% of the participants were overweight or even obese. Moreover, 38% were smokers. None of the participants were taking any medication, and 4% were taking supplements, but none of them took vitamin D supplementation over the last three months prior to this study.

### 2.2. Biochemical Parameters

#### 2.2.1. Ca^2+^, PO_4−_ and PTH

**Figure 1:**
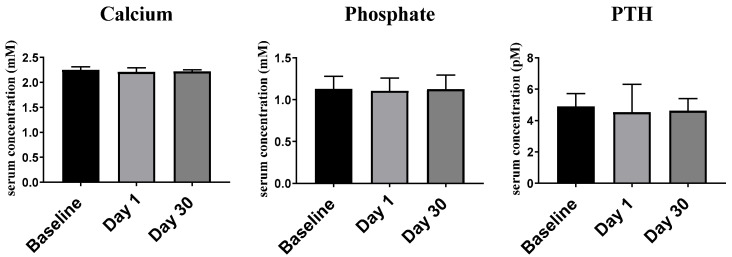
Serum concentration of Ca^2+^, PO_4−_ and PTH. The median serum concentrations were compared between days 0, 1 and 30 of vitamin D_3_ supplementation. The error bars represent interquartile range. Data were analyzed by non-parametric repeated measure ANOVA followed by Wilcoxon signed-rank multiple comparison test.

For serum Ca^2+^ levels, the median as well as 25th and 75th percentiles were, at day 0 (baseline): 2.21 (2.18–2.31) mM, at day 1, 2.21 (2.17–2.29) mM, and at day 30, 2.22 (2.13–2.25) mM (Figure 1). No statistically significant differences between the days of measurement were observed (*p* > 0.05). Similarly, the vitamin D_3_ bolus resulted, neither for serum PO_4−_ (day 0: 1.13 (0.98–1.28) mM, day 1: 1.11 (0.99–1.26) mM and day 30: 1.13 (0.90–1.29) mM) nor for PTH (day 0: 4.90 (3.95–5.72) pM, day 1: 4.53 (3.79–6.30) pM and day 30: 4.63 (3.30–5.40) pM), in any significant differences (*p* > 0.05).

#### 2.2.2. Lipid Profile

**Figure 2:**
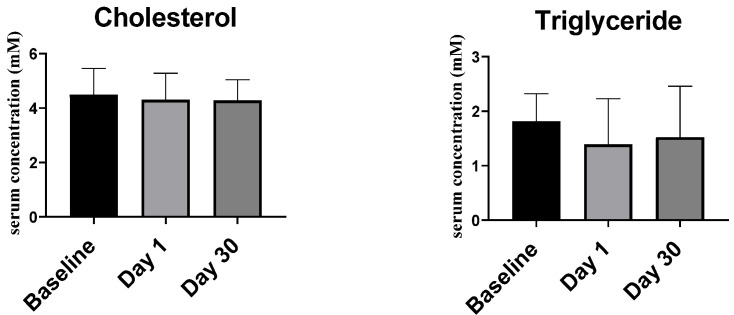
Serum cholesterol and triglyceride concentrations. The mean serum cholesterol and triglyceride concentrations at baseline, day 1 and day 30 are displayed. The error bars represent standard error of mean (SEM) and interquartile range, respectively. Data were analyzed by one-way (ANOVA) followed by Tukey’s multiple comparison and by non-parametric repeated measure ANOVA followed by Wilcoxon signed-rank multiple comparison test, respectively.

Concerning the serum lipid profile (Figure 2), no significant difference was observed for cholesterol concentrations at day 0 (4.49 ± 0.14 mM), day 1 (4.31 ± 0.140 mM) and day 30 (4.28 ± 0.11 mM), nor for triglyceride levels at day 0 (1.82 (1.14–2.33) mM), day 1 (1.39 (1.09–2.23) mM) and day 30 (1.52 (1.15–2.46) mM).

#### 2.2.3. Serum Vitamin D

**Figure 3:**
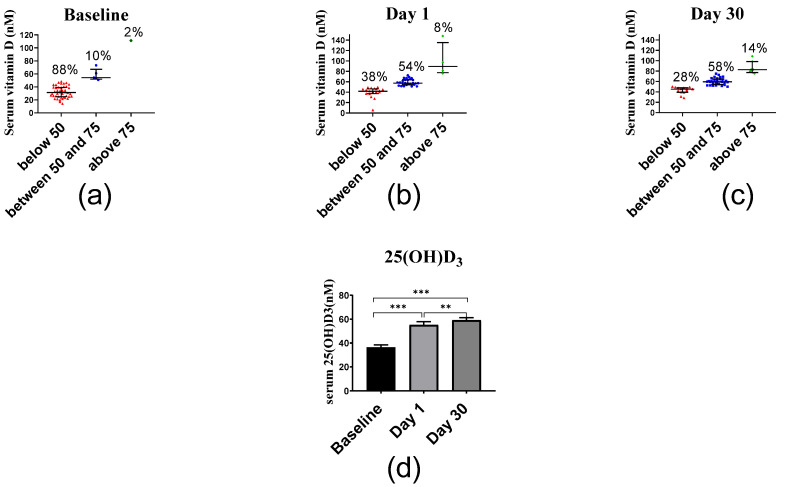
Serum 25(OH) D_3_ concentrations before and after vitamin D_3_ supplementation. (**a**) Baseline levels, (**b**) day 1 levels, and (**c**) day 30 levels. (**d**) Columns represent medians of serum 25(OH)D_3_, and error bars indicate interquartile range. Data were analyzed by non-parametric repeated measure ANOVA followed by Wilcoxon signed-rank multiple comparison test. *** *p* < 0.001, ** *p* < 0.01.

At baseline, most study participants had serum vitamin D levels less than 50 nM, i.e., they were vitamin D deficient (Figure 3). Compared to baseline (33.05 (25.40–43.30) nM), the 25(OH)D_3_ levels at day 1 (53.95 (44.70–63.93) nM) and day 30 (58.15 (49.03–66.93) nM) increased significantly (baseline vs. day 1, *p* < 0.001, baseline vs. day 30, *p* < 0.001 and day 1 vs. day 30, *p* < 0.01).

#### 2.2.4. Inflammatory Markers

**Figure 4:**
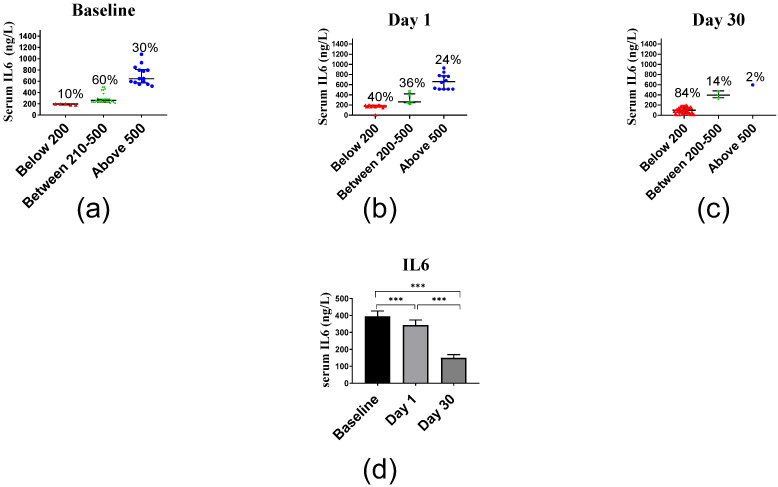
Serum IL6 levels before and after vitamin D supplementation. (**a**) Baseline levels, (**b**) day 1 levels, and (**c**) day 30 levels. (**d**) Columns represent the median serum IL6 concentration at baseline, day 1 and day 30, and error bars indicate interquartile range. Data were analyzed by non-parametric repeated measure ANOVA followed by Wilcoxon signed-rank multiple comparison test. *** *p* < 0.001.

The average IL6 serum levels of 394.83 ng/L at baseline decreased at day 1 to 342.70 ng/L and at day 30 even to 149.83 ng/L (Figure 4). Median and interquartile of IL6 levels at days 0, 1 and 30 were 278.83 (228.83–545.50) ng/L, 245.50 (178.83–487.83) ng/L and 112.17 (58.00–178.83) ng/L, respectively. The decrease in IL6 levels between baseline vs. day 1 (*p* < 0.001), baseline vs. day 30 (*p* < 0.001) and day 1 vs. day 30 (*p* < 0.001) was significant.

**Figure 5:**
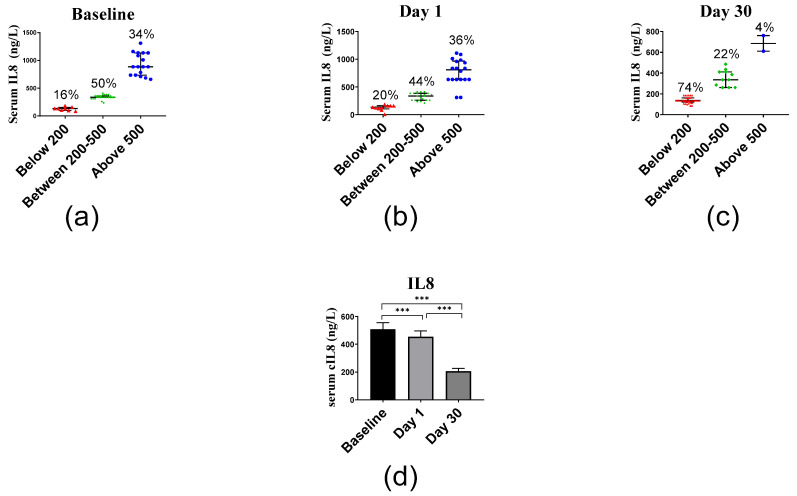
Serum IL8 levels before and after vitamin D supplementation: (**a**) baseline levels, (**b**) day 1 levels, and (**c**) day 30 levels. (**d**) Columns represent the median serum IL6 concentration at baseline, day 1 and day 30, and error bars indicate interquartile range. Data were analyzed by non-parametric repeated measure ANOVA followed by Wilcoxon signed-rank multiple comparison test, and *p* values are shown where the difference between days was determined to be statistically significant. *** *p* < 0.001.

The average IL8 serum levels of 508.38 ng/L at baseline decreased at day 1 to 443.90 ng/L and at day 30 even to 206.90 ng/L (Figure 5). Median and interquartile range of IL8 levels were 360.75 (310.75–748.00) ng/L, 360.75 (235.75–635.75) ng/L and 135.75 (134.50–260.75) ng/L at days 0, 1 and 30, respectively, and differed significantly (*p* < 0.001).

**Figure 6:**
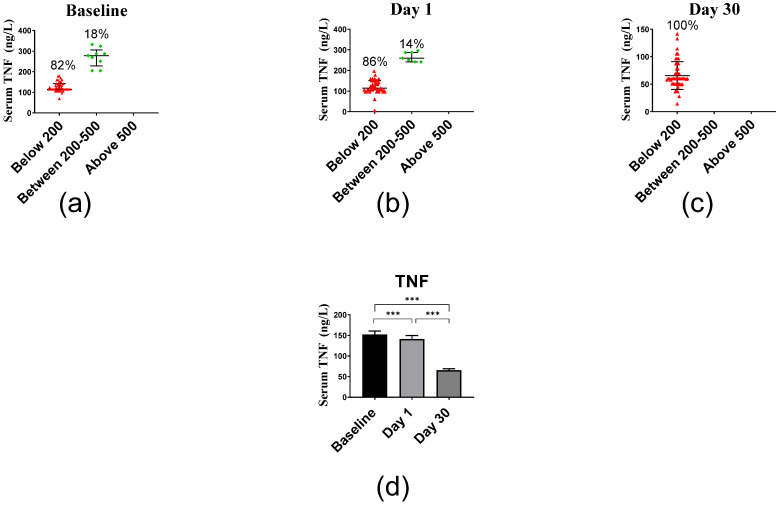
Serum TNF levels before and after vitamin D supplementation. (**a**) Baseline levels, (**b**) day 1 levels, and (**c**) day 30 levels. (**d**) Columns represent the median of TNF concentrations, and error bars indicate interquartile range. Data were analyzed by non-parametric repeated measure ANOVA followed by Wilcoxon signed-rank multiple comparison test. *** *p* < 0.001.

The average TNF serum levels of 151.95 ng/L at baseline decreased at day 1 to 141.37 ng/L and at day 30 even to 65.62 ng/L (Figure 6). Median and interquartile range of TNF levels were 123.55 (114.45–162.18) ng/L, 123.55 (105.36–159.91) ng/L and 59.91 (49.91–78.09) ng/L at days 0, 1 and 30, respectively. The decrease in IL6 levels was significant between baseline vs. day 1 (*p* < 0.001), baseline vs. day 30 (*p* < 0.001) and day 1 vs. day 30 (*p* < 0.001).

The relationships between inflammatory markers were determined by calculating the correlation coefficients between their serum levels (Table 2). There was a significant positive correlation between IL6 and IL8 concentrations at baseline, day 1 and day 30 and significant positive correlation between IL6 and TNF as well as IL8 and TNF concentrations at baseline and day 1. There were no significant correlations between IL6 and TNF nor IL8 and TNF levels at day 30.

## 3. Discussion

The participants of this study are representative of the general population in Saudi Arabia. BMI ranged between 20 and 39 kg/m^2^ (27.5 ± 4.7), indicating that most individuals were overweight or even obese. This may be due to reduced outdoor activities and lack of interest in participating in physical activities due to warm weather in Saudi Arabia. Moreover, it parallels the observation that the prevalence of obesity has increased dramatically in many countries around the world [29]. Saudi Arabia has become increasingly westernized in recent decades and today has one of the highest prevalence of overweight and obesity [30]. This increase in average BMI has contributed to the development of other diseases, such as diabetes, high blood pressure, osteoarthritis, cardiovascular disease and certain types of cancers [31]. Furthermore, the participants were characterized by an average low vitamin D status, as is often observed with obese people [32].

In the present study, we observed baseline serum levels of the cytokines IL6, IL8 and TNF of 395, 508 and 152 ng/L, respectively. This represents an extreme release of pro-inflammatory cytokines [33]. Previous studies have suggested a strong positive correlation between obesity and pro-inflammatory markers, partly reflecting the fact that adipocytes and their associated immune cells are the source of many pro-inflammatory adipokines [34]. Furthermore, studies have found that the increased release of cytokines and decreased adiponectin levels play an essential role in the pathogenesis of obesity [35]. Vgontzas et al. [36] demonstrated elevated levels of both TNF and IL6 in obese subjects, smokers and type 2 diabetes patients. Obesity results in the increased secretion not only of TNF but also of resistin, IL1β and IL6 [35,37]. Thus, this study confirms that overweight and obese people are characterized by increased levels of inflammatory markers.

Inflammation is a crucial etiology in the development of chronic diseases, such as heart diseases, metabolic syndrome and type 2 diabetes [38]. Moreover, during the COVID-19 pandemic, vitamin D deficiency was linked to cytokine storms and was positively correlated with the severity of the disease [39]. The definition of recommended 25(OH)D_3_ serum levels is still under debate, but the majority of researchers in the vitamin D field as well as medical laboratories consider a serum level of 30 ng/mL (75 nM) as a critical threshold [40]. Below this level, a person is considered vitamin D insufficient or even deficient (below 20 ng/mL (50 nM)). The present results are in agreement with previous studies conducted in Saudi Arabia [41,42]. Epidemiological data show that vitamin D deficiency is dominant in 30% of males and 35% of females [43,44]. This observation may be related to inadequate exposure to sunlight, inadequate consumption of dairy products, skin photo-type and reduced outdoor activities [45].

The present research demonstrates that a single bolus of vitamin D3 (80,000 IU) changes the vitamin D status from deficient to more normal levels. Approximately 76% of the participants gained serum vitamin D levels of 50 nM and above after one month (day 30). However, a different response was observed where the participants can be divided into low, mid and high responders [46,47]. Accordingly, high and mid responders may already be sufficiently supplemented with a single vitamin D_3_ bolus, while low responders may need a monthly bolus treatment for a longer time, in order to reach an optimal concentration of higher than 100 nM.

Since vitamin D_3_ is a key regulator of Ca^2+^ and PO_4−_ homeostasis and controls in an antagonistic fashion, the peptide hormone PTH being important for bone metabolism [48], it is important to test whether the vitamin D_3_ bolus applied in this study affects respective serum levels. A dose of 80,000 IU vitamin D_3_ given once to the participants resulted in no significant differences in Ca^2+^, PO_4−_ and PTH serum concentrations at any of the studied three time points. These results are supported by the findings of Kearns et al. [49] and suggest that the vitamin D_3_ bolus was safe.

In the current study, a single vitamin D_3_ bolus reduced serum concentrations of IL6. This observation is in agreement with the cohort study on Irish adults conducted by Laird et al. [50], who observed significant negative correlations between vitamin D and inflammatory markers such as IL6 and C-reactive protein (CRP). Moreover, several in vitro studies indicated that 1,25(OH)_2_D_3_ inhibits the secretion of IL6 in different cell types. The present findings are in agreement with Hashemi et al., who reported reduced IL17A and IL6 levels and increased IL10 levels in mRNA expression in multiple sclerosis patients after vitamin D_3_ supplementation [51]. IL6 levels are upregulated during inflammation of the central nervous system, which results in neuronal damage, especially in axons [52]. Vitamin D_3_ supplementation was found to improve multiple sclerosis by downregulation of both IL17A and IL6 levels [51]. Furthermore, a downregulation of IL6 levels was found after vitamin D_3_ supplementation in type 2 diabetic patients [53].

Another finding of this study was the significant reduction of TNF concentrations. The present findings agree with in vitro studies where vitamin D reduced the synthesis and secretion of TNF [54,55]. In addition, several clinical trials with patients suffering from chronic diseases have reported a positive effect of vitamin D on reducing TNF levels [56]. Collectively, the present data confirmed that vitamin D affects the TNF signaling pathway. The most significant changes occurred after 30 days, which may suggest a long-term effect of a single high dose of vitamin D_3_ supplement. Vitamin D_3_ bolus supplementation also reduced IL8 serum levels. This observation is supported by Dauletbaev et al. [57], who examined the ability of vitamin D to downregulate IL8 production in macrophages. Vitamin D upregulates the transcription of the anti-inflammatory gene *DUSP1* (dual specificity phosphatase 1), which partly controls the production of the inflammatory chemokine IL8 [57]. In general, the association between 25(OH)D_3_ serum concentration with multiple inflammatory markers including IL6, IL8 and TNF has been addressed in numerous human studies in diseased subjects [58,59]. In contrast, only a few studies have been conducted with healthy subjects addressing the possible relation between vitamin D and inflammatory markers [60]. For example, a study accomplished in Kuwait on younger females (aged 19–47) analyzed a wider spectrum of cytokines (IL1β, IL6, IL8, IL7, IFNγ, TNF, IL4, IL10 and IL13) but only reported an inverse correlation between 25(OH)D_3_ serum levels with TNF and IL8 concentration, while CRP levels were elevated [61]. The strong positive correlation between the inflammatory markers IL6, IL8 and TNF may indicate an interrelationship between their production. Taken together, these results indicate that proinflammatory cytokines interact and that a single high dose of vitamin D_3_ is able to reduce these inflammatory markers.

## 4. Materials and Methods

### 4.1. Study Design

Fifty healthy Saudi males aged between 18 and 60 were recruited from the staff of King Abdul Aziz University and King Fahd Medical Research Center and their families. This study was conducted from January to December 2019. The study protocol was approved by the King Abdulaziz University Hospital Ethics Committee (reference no. 30-18, 5 February 2018). All procedures were conducted in compliance with the institution’s ethical guidelines. All participants gave their written informed consent prior to entering the study.

The selection criteria were based upon the following: no intake of vitamin D_3_ supplement for at least the past three months. Apart from this, individuals suffering from systemic diseases including chronic liver or chronic kidney diseases were excluded.

After overnight fasting, blood samples were collected (day 0) to determine the baseline levels of the biochemical variables. Then, a high dose of vitamin D_3_ (80,000 IU) was given orally to all participants only once. After 24 h, blood samples were collected again (day 1). No further vitamin D_3_ supplementation was given to the participants. The third set of blood samples was collected after one month (day 30).

### 4.2. Questionnaire and Anthropometric Measurements

The questionnaire was designed after consultation with experts in this field, in order to collect general demographic information and their medical history. Since several health problems may affect vitamin D digestion, absorption, metabolism and excretion, the participants were questioned especially about liver and kidney diseases. Other questions regarded the usage of vitamin D_3_ supplementation, multivitamins, including minerals such as Ca^2+^. One category was used to describe the smoking habits of the participants. The demographic features that were assessed were sex, age, weight, BMI, waist circumference, hip circumference and WHR. Subjects were indicated as having abdominal fat accumulation if their WHR > 0.9.

### 4.3. Serum Specimen

Blood samples were collected into a plain tube and allowed to clot for 30 min at room temperature. Afterward, samples were then centrifuged at 3000 rpm for 10 min to separate the serum. To avoid freezing and thawing, the sera were divided into aliquots and kept at −80 °C until further analysis. The vitamin D status was determined by using CIMA-based kit (Abbott, Sligo, Ireland). The lower detection limit was 3.4 ng/mL (8.5 nM). The inflammatory cytokines were measured using ELISA kits (BT Lab, Birmingham, United Kingdom). All biochemical variables were measured using fully automated systems in the chemistry lab at King Abdulaziz University Hospital. 

### 4.4. Statistical Analysis

Analyses of the data were conducted using Statistical package for Social Science (SPSS) version 23.0 for windows (SPSS Inc., Chicago, IL, USA), whereas GraphPad Prism 7 software was used to represent graphs. Characteristics of study participants were reported as means and SD. Variables specified with measurement were summarized as SD and medians with 25th and 75th percentiles as well as minimum and maximum values were analyzed by nonparametric tests. Nonparametric tests were reported as median with 25th and 75th percentiles and were used because the assumptions for one-way ANOVA and *t* tests were not met. Friedman’s ANOVA (F) was applied to compare more than two dependent (related) sets of days after vitamin D supplementation; then, Wilcoxon signed-rank (W) test equivalent to the paired *t* test was used to compare each two pairs of days. Correlations were carried out using Spearman’s correlation (r). Results were considered significant if *p* < 0.05.

## 5. Conclusions

The present study shows the positive short- and long-term effects on improving inflammatory markers in a population of healthy Saudi males (*n* = 50) after the intake of a single oral vitamin D_3_ bolus. The population is characterized by high serum concentrations of IL6, IL8 and TNF, particularly in those individuals with high BMI. Therefore, the present results may have an important implication on future recommendations for vitamin D_3_ supplementation, for instance, taking only one vitamin D_3_ bolus per month.

Nevertheless, further studies should include larger population sizes (*n* > 50) of male and female participants in cohorts (e.g., married/unmarried, employed/unemployed, high/low education) from all over Saudi Arabia to guarantee the representativeness of the study and to confirm the validity of the present results. This also includes cellular and molecular studies that may reveal the underlying mechanisms of the observed effects.

## Figures and Tables

**Table 1:** Demographic characteristics of studied participants. SD, standard deviation.

	Mean	SD	Range
**Age (years)**	30.3	±6.2	18–53
**Age N (%)**	All participants (*n* = 50)		
**20 to less than 40 years**	47 (94)		
**40 to less than 60 years**	3 (6)		
**60 years**	0 (0)		
	Mean	SD	Range
**Height (cm)**	174.3	±6.2	160–188
**Body weight (kg)**	83.8	±15.2	53–121
**BMI (kg/m^2^)**	27.6	±4.7	20.1–39.1
**BMI classification**	All participants (*n* = 50)		
**Underweight**	0 (0)		
**Normal (18.5–24.99)**	17 (34%)		
**Overweight (25–29.99)**	16 (32%)		
**Obese (>30)**	17 (34%)		
	Mean	SD	Range
**Waist circumference (cm)**	94.4	±14.8	59–126
**Hip circumference (cm)**	104.9	±15.7	39–128
**WHR**	0.91	±0.13	0.67–1.51
**Health status N (%)**	All participants (*n* = 50)		
**Liver disease**	0 (0%)		
**Kidney diseases**	0 (0%)		
**Healthy**	50 (100%)		
**Taking any medicines N (%)**	All participants (*n* = 50)		
	Yes	0 (0)	
	No	50 (100%)	
**Broken any bones N (%)**			
	Yes	35 (70%)	
	No	15 (30%)	
**Taking any supplements N (%)**			
	Minerals	2 (4%)	
	Vitamin D	0 (0%)	
	Ca^2+^	0 (0)%	
	Nothing	48 (96%)	
**Awareness to vitamin D (if taking test of vitamin D concentration) N (%)**			
	Yes	15 (30%)	
	No	35 (70%)	
**Sun exposure N (%)**			
	Less than 5 min/day	1 (2%)	
	5–15 min/day	7 (14%)	
	15–30 min/day	12 (24%)	
	More than 30 min/day	30 (60%)	
**Exercise rate N (%)**			
	Always	15 (30%)	
	Usually	30 (60%)	
	Rarely	5 (10%)	
**Smoke status N (%)**			
	Smoker	19 (38%)	
	Non-smoker	31 (62%)	

**Table 2:** Spearman correlation coefficients (r) between inflammatory markers.

**Inflammatory** **Markers**	**Baseline, IL6** ***n* = 50**		**Day 1, IL6** ***n* = 50**		**Day 30, IL6** ***n* = 50**	
	r	*p*	r	*p*	r	*p*
IL8	0.50 **	<0.01	0.51 **	<0.01	0.54 **	<0.01
TNF	0.64 **	<0.01	0.56 **	<0.01	0.03	0.82
**Inflammatory** **Markers**	**Baseline, IL8** ***n* = 50**		**Day 1, IL8** ***n* = 50**		**Day 30, IL8** ***n* = 50**	
	r	*p*	r	*p*	r	*p*
TNF	0.50 **	<0.01	0.43 **	<0.01	0.05	0.74

IL6 and IL8, IL6 and TNF as well as IL8 and TNF concentrations at baseline, day 1 and day 30. ** *p* < 0.01.
